# Editorial: Advances in the Prevention and Treatment of Inflammation-Associated Preterm Birth

**DOI:** 10.3389/fimmu.2016.00264

**Published:** 2016-06-30

**Authors:** Jeffrey A. Keelan, John P. Newnham

**Affiliations:** ^1^School of Women’s and Infants’ Health, The University of Western Australia, Perth, WA, Australia

**Keywords:** preterm birth, inflammation, infection, fetus, oxidative stress, anti-inflammatory agents, antimicrobials, probiotics

**The Editorial on the Research Topic**

**Advances in the Prevention and Treatment of Inflammation-Associated Preterm Birth**

Despite the widely appreciated fact that preterm birth (PTB) is a syndrome and the potential consequence of many different pathways (1), there is now unequivocal evidence that inflammation lies at the core of the majority of the pathophysiological causes of PTB. Inflammation within the amniotic cavity – characterized by chorioamnionitis and/or elevated levels of amniotic fluid cytokines and chemokines – is the major driver of preterm labor less than 34 weeks gestation and an important contributor to later preterm deliveries (2–4). However, the causes of the inflammation and the strategies by which it can be safely and effectively prevented and treated are less clear and the subject of ongoing investigation and debate. While ascending bacterial infection is a particularly important and well-studied cause of inflammation-associated PTB in very preterm deliveries, other “sterile” causes are dominant at later gestational ages. The nature, localization, timing, and extent of the inflammatory insult likely determine the obstetric outcome and degree of risk to the fetus. These factors also dictate possible pharmacotherapeutic approaches that might be employed to achieve better pregnancy outcomes – namely, minimal neonatal morbidity and optimal long-term health and development of the child.

In this research topic, we have invited contributions from a range of internationally recognized clinicians and scientists relating to inflammation-associated PTB from both the causal and therapeutic perspectives. To place the discussions in the broader clinical context, Newnham et al. describe the problem of PTB and major intervention strategies that are being applied in clinics around the world to prevent PTB. They outline the evidence supporting efficacy and safety, and the likely impact of clinical implementation on PTB rates. They contend that we are now in a position to translate research into clinical care, although such interventions will require integration and coordination of multidisciplinary teams, tailored to different communities and resource settings. Next, Kemp discusses the immunological factors involved in pathogenesis of fetal inflammatory response syndrome (FIRS), with special emphasis on the role of pattern recognition receptors, defensins, and complement activation. He reviews, in detail, the evidence for a fetal contribution to the overall intrauterine inflammatory response to microbial infection and its significance with respect to neonatal sequelae.

Following on, Payne and Bayatibojakhi outline the roles played by different microorganisms (bacteria, fungi, yeasts, and viruses) in the process of infection-driven PTB and the contribution of microbiome studies to our understanding of this topic. They reiterate the important point that intra-amniotic infection is frequently a polymicrobial disease and discuss technical aspects related to the generation and interpretation of microbiome data – in particular the “disconnect” between DNA-based identification and presence of viable microorganisms. The recent study by Prince et al. (5) further contributes to this topic, providing novel links between the microbiome and the metabolome in preterm deliveries. Continuing with the microbial-inflammation theme, Ireland and Keelan move the focus to the maternal response to infection and the serological response to *Ureaplasma*, in particular. While this intracellular microorganism is known to be commonly associated with inflammation-driven PTB, the relatively low incidence of infection-associated PTB in the presence of high vaginal *Ureaplasma*-colonization rates (~50% in pregnancy) remains unexplained. This review explores the role of the maternal host response to colonization in determining risk and pregnancy outcomes. Next, Menon overviews the role of oxidative stress as a driver of PTB and preterm prelabor rupture of membranes (PPROM). He examines the evidence supporting the contribution of reactive oxygen species (ROS) to the pathogenesis of PPROM and PTB and the pregnancy conditions that may give rise to ROS generation and downstream adverse effects: MAPK activation, telomere reduction, DNA damage, senescence, and apoptosis. He suggests that the release of secreted senescence biomarkers may play a key role in the triggering of PTB and PPROM and, as such, are targets for new interventional strategies. A recent publication from his group reinforces the theme of this review (6).

From inflammatory pathophysiology we move to therapy. Ng et al. commence by exploring the potential of novel anti-inflammatory drugs to block inflammatory signaling and prevent the release of cytokines and other mediators that trigger labor and delivery and cause inflammation-associated fetal morbidity. They discuss the efficacy of a range of compounds and then discuss the barriers to clinical translation and the challenges of adopting such anti-inflammatory strategies in different pregnancy scenarios. Next, Yang et al. examine the potential application of administration of probiotics to prevent PTB. They initially discuss the evidence supporting an association between altered vaginal microbiome and adverse pregnancy outcome, looking at the relationship between the presence of different *Lactobacillus* species and PTB rates, in different populations. They, then, review the evidence from their own studies (and others) on the properties and applications of probiotics, such as *Lactobacillus rhamnosus* GR-1, for preventing PTB. They conclude that the strategy holds promise, but large scale clinical trials are required with appropriate power to demonstrate the benefits and risks. A recent trial of oral probiotics (*L. rhamnosus* GR-1 plus *L. reuteri* RC-14) supports the potential effectiveness of the approach in terms of normalizing vaginal microbiota (7).

Lamont, then, reviews the evidence around the use of antibiotics for the prevention of PTB, looking at the results of clinical trials and meta-analyses and pointing to errors in their design and conclusions. He stresses the importance of applying the right therapy at the right time to the right cohort of patients and points to the success of studies using clarithromycin early in pregnancy to treat women with abnormal vaginal microbiota. Finally, Keelan et al. discuss the potential applications of a new macrolide antibiotic, solithromycin, in the prevention and treatment of pregnancy infections and PTB. Drawing on their own studies in human placentas and the pregnant sheep model, combined with other data on antimicrobial efficacy, they discuss the potency of solithromycin against the organisms that typically infect the amniotic cavity. This review also highlights solithromycin’s unique ability to cross the placenta and treat the infection at its source following oral maternal administration.

## Author Contributions

Both authors are editors of the research topic and contributed to the writing and editing of the editorial.

## Conflict of Interest Statement

The authors declare that the research was conducted in the absence of any commercial or financial relationships that could be construed as a potential conflict of interest.
